# Performance of the MeltPro TB assay as initial test for diagnosis of pulmonary tuberculosis with drug-resistance detection

**DOI:** 10.1186/s10020-023-00743-1

**Published:** 2023-11-07

**Authors:** Zhi-bin Liu, Li-ping Cheng, Hong-qiu Pan, Xiao-cui Wu, Fu-hui Lu, Jie Cao, Lei Wang, Wei Wei, Hong-yu Chen, Wei Sha, Qin Sun

**Affiliations:** 1 Shanghai Clinical Research Center for Infectious Disease (Tuberculosis), Shanghai Key Laboratory of Tuberculosis, Shanghai Pulmonary Hospital, School of Medicine, Tongji University, Shanghai, 200433 China; 2 Department of Clinical Laboratory, Shanghai Pulmonary Hospital, School of Medicine, Tongji University, Shanghai, China; 3 Department of Tuberculosis, The Third People’s Hospital of Zhenjiang, School of Medicine, Jiangsu University, Jiangsu, China

**Keywords:** MeltPro TB, Xpert MTB/RIF, Pulmonary tuberculosis, Drug-resistance, Diagnosis

## Abstract

**Background:**

The MeltPro TB assay (MeltPro) is a molecular rapid diagnostic test designed for detecting resistance to antituberculosis drugs. However, the performance of MeltPro as an initial diagnostic test for simultaneously detecting the presence of *Mycobacterium tuberculosis* (MTB) and drug resistance has not been evaluated. This study aims to assess the performance of MeltPro as initial diagnostic test for simultaneous detection of MTB and drug resistance in clinical samples from patients with presumptive pulmonary tuberculosis (PTB).

**Methods:**

A retrospective analysis was conducted on 1283 patients with presumptive PTB from two clinical centers, out of which 875 were diagnosed with PTB. The diagnostic accuracy of MeltPro, Xpert MTB/RIF (Xpert), and MGIT 960 for PTB detection was evaluated. Rifampicin (RIF), isoniazid (INH), ethambutol (EMB), streptomycin (STR), and fluoroquinolone (FQ) resistance were detected using MeltPro, with Xpert and/or the broth microdilution plate method (MYCOTB) results as references.

**Results:**

For the diagnosis of PTB, MeltPro showed a sensitivity of 69.0%, which was similar to Xpert (72.7%; *P* > 0.05) and higher than MGIT (58.1%; *P* < 0.001). The specificity of MeltPro was 97.1%, similar to Xpert (98.0%; *P* > 0.05). In smear-negative patients, MeltPro's sensitivity was 50.9%, similar to Xpert (56.5%; *P* > 0.05), and higher than MGIT (33.1%; *P* < 0.001). Based on Xpert and/or MYCOTB results, MeltPro exhibited a sensitivity and specificity of 98.3% and 99.2%, respectively, for detecting RIF resistance. Based on MYCOTB results, MeltPro's sensitivity for detecting resistance to INH, EMB, STR, and FQ was 96.4%, 89.1%, 97.5%, and 90.3%, respectively, with specificities of 96.0%, 96.0%, 95.2%, and 99.4%, respectively.

**Conclusion:**

The MeltPro TB assay could potentially be an effective alternative as the initial test for rapid diagnosis of PTB with drug-resistance detection in clinical practice.

## Introduction

Globally, tuberculosis (TB) remains a significant public health challenge, with an estimated 10.6 million people developing TB in 2021, and 4.2 million cases going undiagnosed and unreported, particularly in the context of drug-resistant TB (WHO 2022a). Effective TB management relies on prompt diagnosis of TB and timely detection of drug resistance to initiate appropriate treatment regimens promptly (WHO 2015). Although smear microscopy is widely used for rapid detection of acid-fast bacilli (AFB), it cannot differentiate between nontuberculous mycobacteria and *Mycobacterium tuberculosis* (MTB) (Wei et al. 2023), nor can it distinguish drug-resistant strains from drug-susceptible strains. Culture, considered the gold standard for confirming TB, is not used as a primary diagnostic test in many high-burden TB countries due to cost, infrastructure requirements (biosafety level 3), and the prolonged time required for results (3–6 weeks) (Votintseva et al. 2017). Hence, there is a need for access to fast and accurate detection tests and rapid and accurate drug-susceptibility testing (DST).

World Health Organization (WHO) recommends molecular rapid diagnostic tests to be made available to all individuals with signs or symptoms of TB (WHO 2016). Consequently, Xpert MTB/RIF (Xpert), Xpert MTB/RIF Ultra (Xpert Ultra), Truenat MTB, MTB Plus and MTB-RIF Dx assays (Truenat), and moderate complexity automated nucleic acid amplification tests (NAATs) are recommended as initial tests for diagnosis of TB with drug-resistance detection (WHO 2021c). However, WHO guideline recommends universal access to DST: rapid testing for at least RIF resistance in all patients with bacteriologically confirmed TB, and further testing for FQs and second-line injectable agents in TB patients with RIF resistance (WHO 2016), but unfortunately, Xpert, Xpert Ultra, and Truenat can only detect resistance to rifampicin (RIF), and moderate complexity automated NAATs can only detect resistance to RIF and isoniazid (INH) (WHO 2021b).

The MeltPro TB assay (MeltPro) is a molecular rapid diagnostic test designed for detecting resistance to anti-TB drugs, including RIF, INH, FQs, and second-line injectable agents, etc. The underlying principles of MeltPro rest upon fluorescence PCR melting curve analysis. Within the same self-enclosed test unit, introducing the crude DNA of MTB into the PCR mixtures which contain self-quenched fluorescence probes, conducting PCR amplification and monitoring the real-time fluorescence signals released when the amplified single-stranded DNA bind to the probes, plotting the melting curves based on the negative derivative of fluorescence against temperature to determine the melting temperature (Tm) values, and identifying drug-resistance mutations according to the extent of Tm values decreases, since the Tm value of the wild-type gene exhibits the highest peak. In theory, it can serve as an initial diagnostic test for simultaneously detecting the presence of MTB and drug resistance.

In this study, we evaluated the performance of MeltPro as an initial diagnostic test for simultaneously detecting the presence of MTB and resistance to RIF, INH, FQ, ethambutol (EMB), and streptomycin (STR) in clinical specimens, including sputum, BALF, and pulmonary tissue puncture fluid, from patients with presumptive pulmonary tuberculosis (PTB). The results provide valuable insights into the potential scalability of this assay in clinical practice.

## Materials and methods

### Study design and participants

**Fig. 1:**
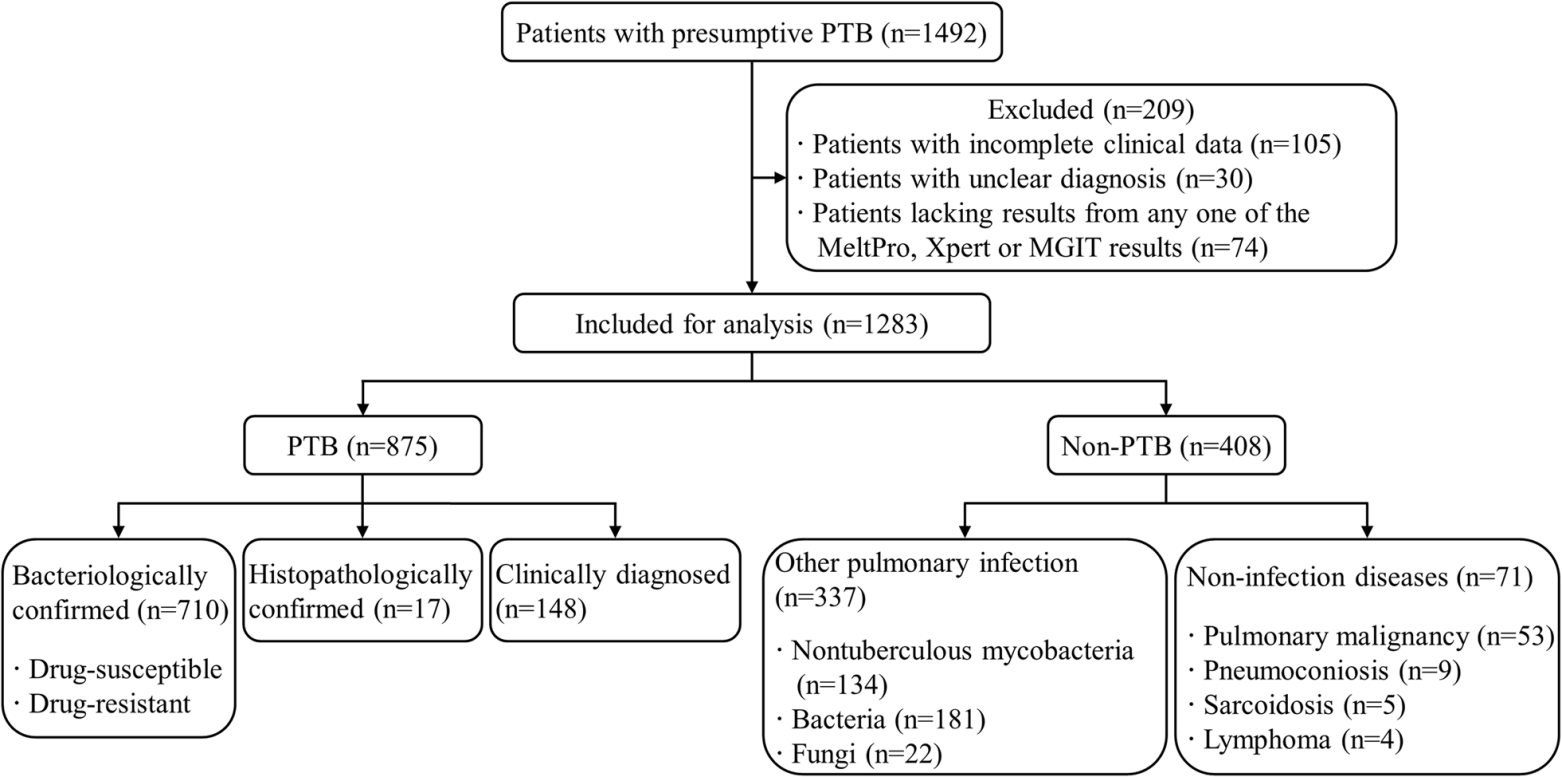
Flow diagram illustrating the process of case enrollment, inclusion, and classification

A total of 1492 patients with presumptive PTB were enrolled in this retrospective study between 1 January 2022 and 31 December 2022. Among them, 1174 patients were from Shanghai Pulmonary Hospital affiliated to Tongji University, and 318 patients were from the Third People’s Hospital of Zhenjiang affiliated to Jiangsu University. After applying specific criteria, 1283 patients were included in the analysis. The inclusion criteria comprised: (1) individuals aged between 16 and 75 years, without sex restrictions; (2) individuals with negative HIV results; and (3) individuals presenting pulmonary lesions consistent with active PTB on imaging, such as patchy opacity, consolidation, lobar infiltration, caseous lesions, multiple nodules, tuberculoma or solitary cavities, multiple cavities, fibrous thick-wall cavity, bronchial dissemination, tree-in-bud signs, with or without calcification. Exclusion criteria encompassed individuals meeting any of the following conditions: (1) incomplete clinical data; (2) unclear diagnosis; or (3) absence of results from any one of the MeltPro, Xpert, and MGIT 960 tests. After applying these criteria, 875 patients were diagnosed with PTB, while 408 patients were diagnosed with non-PTB, as depicted in Fig. 1. Detailed clinical characteristics, including demographic data, chest computed tomography (CT) imaging, specimen types, and laboratory results, were recorded for each included patient. The Ethics Committees of Shanghai Pulmonary Hospital approved this study (Ethics No. K23-254).

### Diagnostic criteria of PTB

According to the WHO Definitions and reporting framework for tuberculosis—2013 revision (updated in December 2014 and January 2020) (WHO 2020), the Official American Thoracic Society/Infectious Diseases Society of America/Centers for Disease Control and Prevention Clinical Practice Guidelines: Diagnosis of Tuberculosis in Adults and Children (Lewinsohn 2017), and the Health Industry Standard of the People's Republic of China—Diagnosis of Pulmonary Tuberculosis (WS 288-2017) released by the National Health Commission of China, the diagnostic criteria of PTB are as follows:

1. Confirmed PTB case: (1) Bacteriologically confirmed PTB case: Patients with positive Mycobacterium culture results and further identification as MTB complex and/or positive results in Xpert test. (2) Histopathologically confirmed PTB case: Patients with negative results in bacteriological tests but who show chronic granulomatous inflammation, with or without caseous necrosis, observed by light microscopy during further histopathological examination, and MTB-DNA-IS6110 is detected in the lesion, with or without positive results for AFB.
2. Clinically diagnosed TB case: Patients with negative results in bacteriological and/or histopathological tests who meet the following three conditions simultaneously: (1) The average diameter of skin scleroma (in PPD skin test with 5 IU) is ≥ 10 mm or there is a positive result in interferon-gamma release assay. (2) Other lung diseases are excluded. (3) Pulmonary lesions show reduction or absorption after anti-TB treatment.

### Specimen collection and preprocessing

Morning sputum (> 2 mL) was collected through deep coughing after gargling with clean water. BALF (> 5 mL) and pulmonary puncture tissue were collected following standard procedures. Each specimen was collected in a tube without deoxyribonuclease, cryopreserved at – 20 °C, and delivered to the laboratory within 24 h. Puncture tissue was ground into homogenized fluid, and the supernatant was transferred to the tube.

A direct smear from sputum, BALF, and puncture fluid was prepared and stained with auramine for examination under light-emitting diode microscopy to detect AFB. The remaining specimen was decontaminated with N-acetyl-L-cysteine and sodium hydroxide (NALC-NaOH) for 15 min, and then neutralized with phosphate buffer saline (PBS). After centrifugation at 3000 × *g* for 20 min, the pellet was resuspended in 3 mL of PBS.

### MeltPro TB assay

The MeltPro TB assay testing was performed in accordance with the manufacturer's instructions. The crude DNA of MTB was extracted from a 1 mL aliquot of the decontaminated specimen using an automatic DNA extraction machine (Zeesan Biotecheh, Xiamen, China) employing the paramagnetic particle method. Subsequently, 5 μL of the crude DNA was introduced into PCR mixtures containing self-quenched fluorescence probes labelled with 6-FAM and 6-TET. PCR amplification and melting curve analysis were carried out using the LightCycler 480 system (Roche Applied Science, Indianapolis, IN, USA). The polymerase chain reaction was conducted as per the following protocol: decontamination for 2 min at 50 °C using uracil-N-glycosylase; denaturation for 5 min at 95 °C; a touchdown program for 10 cycles comprising 10 s at 95 °C, 25 s at 71 °C (with a decrease of 1 °C per cycle), and 30 s at 75 °C, followed by 45 cycles consisting of 10 s at 95 °C, 25 s at 61 °C, and 25 s at 75 °C. Melting curve analysis was initiated with a denaturation step of 2 min at 95 °C, followed by hybridization for 2 min at 40 °C. The temperature was incrementally increased from 40 °C to 85 °C at a rate of 1 °C/step with a 5-s pause between each step. Various mutations in specific gene regions, including the rpoB gene (codons 507-533) for RIF resistance, the ahpC promoter region, inhA94 codon, inhA promoter region, and katG315 codon for INH resistance, the embB gene (codons 306, 368, 378, 380, 406, 497) for EMB resistance, the rrs gene (codons 513–517, 905–908), rpsL43 codon, and rpsL88 codon for STR resistance, and the gyrA gene (codons 88–94) for FQ resistance were detected.

### Xpert MTB/RIF

The Xpert MTB/RIF testing was carried out following the manufacturer's instructions (Cepheid GeneXpert System, Sunnyvale, CA, USA). Briefly, a 1 mL aliquot of the decontaminated specimen was mixed with 2 mL Xpert sample-processing reagent, vortexed for at least 10 s, and incubated at room temperature for 10 min. The mixture was then vortexed for another 10 s and incubated at room temperature for 5 min. A 2 mL aliquot of the mixture was transferred into the Xpert cartridge and loaded into the GeneXpert instrument, and the automatic detection procedure was initiated. The mutations in the rifampicin resistance determining region (RRDR) of the rpoB gene (codons 507–533) were detected.

### MGIT 960 and phenotypic DST

A 0.5 mL aliquot of the decontaminated specimen was added to the mycobacteria growth indicator tube (MGIT) and cultured in the BACTEC MGIT 960 system (Becton, Dickinson and Company, Franklin Lakes, NJ, USA). Positive cultures were confirmed as mycobacteria using Ziehl–Neelsen staining. Further species identification was performed using rho-nitrobenzoic acid (PNB) and Thiophene-2-carboxylic acid hydrazide (TCH) media. Phenotypic DST for the isolates identified as MTB was carried out using the broth microdilution plate method. The critical concentration for RIF, INH, EMB, STR, ofloxacin, and moxifloxacin on the Sensititre MYCOTB plate (Thermo Fisher Scientific, Cleveland, OH, USA) were 0.5 mg/L, 0.125 mg/L, 4 mg/L, 1 mg/L, 2 mg/L, and 0.25 mg/L, respectively (Thermo Scientific 2019a, Thermo Scientific 2019b; WHO 2022b).

### Statistical analysis

Statistical analysis was performed using SPSS 26.0 software (SPSS Inc., Chicago, IL, USA). Continuous variables with a normal distribution were expressed as mean ± standard deviation (SD). The Student's T-test was used to compare the means, and the chi-square test was used for the comparison of categorical data between groups. The diagnostic performance of MeltPro, Xpert, and MGIT for PTB, with a reference standard based on bacteriologically/histopathologically confirmed PTB and clinically diagnosed PTB, and the performance of MeltPro for detection of resistance to RIF, INH, EMB, STR, and FQ using Xpert and/or MYCOTB results as references were calculated. This included sensitivity, specificity, positive predictive value (PPV), and negative predictive value (NPV). Additionally, the receiver operating characteristic (ROC) curve was drawn, and the area under the curve (AUC) was calculated. All tests were two-sided, and a difference was considered statistically significant at *P* < 0.05.

## Results

### Baseline comparison of demographic and clinical characteristics between the PTB and non-PTB groups

Patient characteristics are presented in Table 1. The PTB group comprised 522 males (59.7%) and 353 females (40.3%), with an average age of 42.7 ± 7.3 years. The non-PTB group had 232 males (56.9%) and 176 females (43.1%), with an average age of 44.5 ± 7.4 years. The proportion of patients with a positive QuantiFERON-TB Gold (QFT) test in the PTB group (80.7%) was higher than that in the non-PTB group (29.2%, *P* < 0.01). Moreover, the proportion of patients with diabetes in the PTB group (15.1%) was higher than that in the non-PTB group (5.4%, *P* < 0.01). However, there were no significant differences in sex, age, body mass index, imaging findings, specimen types, and other baseline characteristics between the two groups.

**Table 1:** Baseline comparison of demographic and clinical characteristics between the PTB and non-PTB groups

	PTB (n = 875)	Non-PTB (n = 408)	*P*-value
*Characteristics*			
Sex			0.344
Male, n (%)	522 (59.7%)	232 (56.9%)	
Female, n (%)	353 (40.3%)	176 (43.1%)	
Age, years	42.7 ± 7.3	44.5 ± 7.4	0.263
BMI, kg/m^2^	20.2 ± 2.8	21.1 ± 3.2	0.143
QuantiFERON-TB ( +), n (%)	706 (80.7%)	119 (29.2%)	< 0.01
*Presenting symptoms or signs*			
No special clinical symptoms, n (%)	194 (22.2%)	72 (17.6%)	0.065
Non-productive cough, n (%)	562 (64.2%)	276 (67.6%)	0.231
Productive cough, n (%)	435 (49.7%)	225 (55.1%)	0.072
Chest tightness and/or wheezing, n (%)	177 (20.2%)	98 (24.0%)	0.123
Fever, n (%)	161 (18.4%)	91 (22.3%)	0.113
Fatigue, n (%)	110 (12.6%)	44 (10.8%)	0.407
Thoracalgia, n (%)	93 (10.6%)	52 (12.7%)	0.265
Hemoptysis and/or blood in phlegm, n (%)	92 (10.5%)	49 (12.0%)	0.444
Inappetence, n (%)	59 (6.7%)	19 (4.7%)	0.168
Weight loss, n (%)	47 (5.4%)	18 (4.4%)	0.498
*Radiological characteristics*			
Involvement extent of lung lesions			0.111
≤ 3 lobes, n (%)	621 (71.0%)	307 (75.2%)	
≥ 4 lobes, n (%)	254 (29.0%)	101 (24.8%)	
Cavitary, n (%)	245 (28.0%)	98 (24.0%)	0.134
*Underlying disease*			
COPD, n (%)	43 (4.9%)	23 (5.6%)	0.585
Bronchiectasis, n (%)	66 (7.5%)	37 (9.1%)	0.349
Diabetes, n (%)	132 (15.1%)	22 (5.4%)	< 0.01
Previous history of tuberculosis, n (%)	224 (25.6%)	96 (23.5%)	0.425
*Specimen*			0.521
Sputum, n (%)	657 (75.1%)	312 (76.5%)	
BALF, n (%)	187 (21.4%)	78 (19.1%)	
Pulmonary puncture fluid, n (%)	31 (3.5%)	18 (4.4%)	

*PTB* pulmonary tuberculosis, *BMI* Body mass index, *COPD* chronic obstructive pulmonary disease, *BALF* bronchoalveolar lavage fluid

### Consistency of MeltPro, Xpert, MGIT and smear

**Fig. 2:**
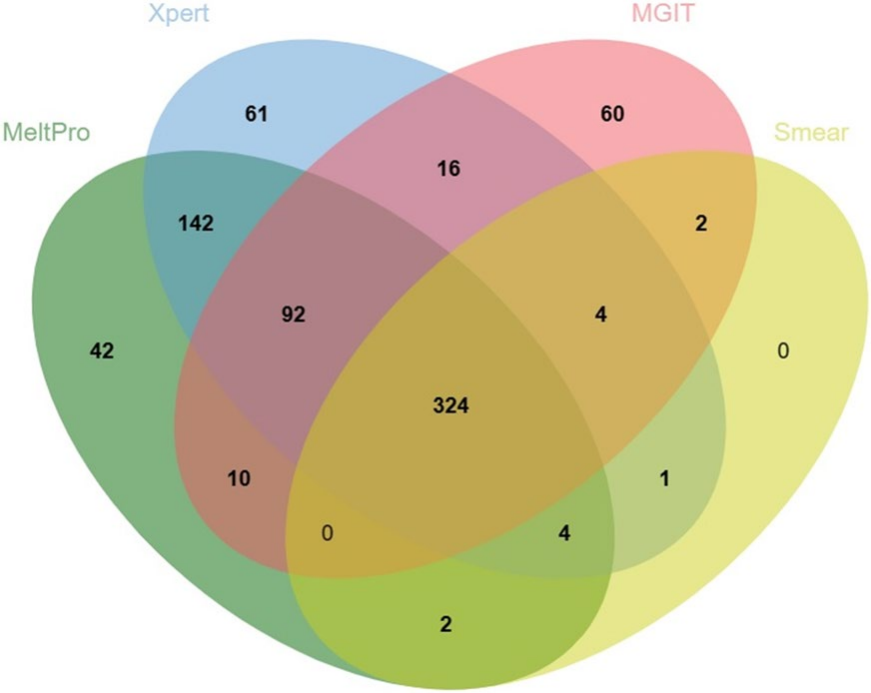
Venn diagram depicting the overlapping test results for diagnosing pulmonary tuberculosis

Among the 1283 included specimens, MeltPro and Xpert showed positive results in 616 and 644 cases, respectively. The false-positive rates based on the final diagnosis were 1.9% (12 cases) for MeltPro and 1.2% (8 cases) for Xpert. The positive results of MeltPro, Xpert, MGIT, and smear among the 875 PTB cases are depicted in Fig. 2.

### Performance and ROC curves of MeltPro, Xpert, MGIT and smear for diagnosis of PTB

The results are summarized in Table 2. The overall sensitivity of MeltPro, Xpert, MGIT, and smear for diagnosing PTB was 69.0%, 72.7%, 58.1%, and 38.5%, respectively. MeltPro showed similar sensitivity to Xpert (*χ*^2^ = 2.834, *P* = 0.092) and higher sensitivity than MGIT (*χ*^2^ = 22.733, *P* < 0.001). The overall specificity of MeltPro, Xpert, MGIT, and smear for diagnosing PTB was 97.1%, 98.0%, 100%, and 88.2%, respectively. In smear-negative patients, the sensitivity of MeltPro, Xpert, and MGIT for diagnosing PTB was 50.9%, 56.5%, and 33.1%, respectively. MeltPro showed similar sensitivity to Xpert (*χ*^2^ = 3.364, *P* = 0.067) and higher sensitivity than MGIT (*χ*^2^ = 35.159, *P* < 0.001).

**Table 2:** Diagnostic performance of the tests for diagnosis of pulmonary tuberculosis

	Sensitivity % (95%CI; n/N)	Specificity % (95%CI; n/N)	PPV %	NPV %
*Total*				
MeltPro	69.0 (65.8–72.1; 604/875)	97.1 (94.8–98.4; 396/408)	98.1	59.4
Xpert	72.7 (69.6–75.6; 636/875)	98.0 (96.0–99.1; 400/408)	98.8	62.6
MGIT	58.1 (54.7–61.3; 508/875)	100 (98.8–100.0; 408/408)	100	52.6
Smear	38.5 (35.3–41.8; 337/875)	88.2 (84.6–91.1; 360/408)	87.5	40.1
*Smear positive*				
MeltPro	97.9 (95.6–99.1; 330/337)	91.7 (79.1–97.3; 44/48)	98.8	86.3
Xpert	98.5 (96.4–99.5; 332/337)	97.9 (87.5–99.9; 47/48)	99.7	90.4
MGIT	97.9 (95.6–99.1; 330/337)	100 (90.8–100.0; 48/48)	100	87.3
*Smear negative*				
MeltPro	50.9 (44.6–55.2; 274/538)	97.8 (95.5–99.0; 352/360)	97.2	57.1
Xpert	56.5 (52.2–60.7; 304/538)	98.1 (95.9–99.2; 353/360)	97.7	60.1
MGIT	33.1 (29.2–37.3; 178/538)	100 (98.7–100.0; 360/360)	100	50.0
*Sputum*				
MeltPro	65.4 (61.7–69.1; 430/657)	97.1 (94.4–98.6; 303/312)	97.9	57.2
Xpert	70.3 (66.6–73.8; 462/657)	98.4 (96.1–99.4; 307/312)	98.9	61.2
MGIT	54.9 (51.1–58.8; 361/657)	100 (98.5–100.0; 312/312)	100	51.3
*BALF*				
MeltPro	81.3 (74.8–86.5; 152/187)	96.2 (88.4–99.0; 75/78)	98.1	68.2
Xpert	80.7 (74.2–86.0; 151/187)	97.4 (90.2–99.6; 76/78)	98.7	67.9
MGIT	69.5 (62.3–75.9; 130/187)	100 (94.2–100.0; 78/78)	100	57.8
*Pulmonary puncture fluid*				
MeltPro	71.0 (51.8–85.1; 22/31)	100 (78.1–100.1; 18/18)	100	66.7
Xpert	74.2 (55.1–87.5; 23/31)	94.4 (70.6–99.7; 17/18)	95.8	68.0
MGIT	54.8 (36.3–72.2; 17/31)	100 (78.1–100.1; 18/18)	100	56.3

*MGIT* mycobacterial growth indicator tube, *BALF* bronchoalveolar lavage fluid, *PPV* positive predictive value, *NPV* negative predictive value

The sensitivity of MeltPro, Xpert, and MGIT in sputum samples was 65.4%, 70.3%, and 54.9%, respectively. MeltPro showed similar sensitivity to Xpert (*χ*^2^ = 3.575, *P* = 0.059) and higher sensitivity than MGIT (*χ*^2^ = 15.122, *P* < 0.001). In BALF samples, the sensitivity of MeltPro, Xpert, and MGIT was 81.3%, 80.7%, and 69.5%, respectively. MeltPro showed similar sensitivity to Xpert (*χ*^2^ = 0.017, *P* = 0.896) and higher sensitivity than MGIT (*χ*^2^ = 6.977, *P* = 0.008). In pulmonary puncture fluid samples, the sensitivity of MeltPro, Xpert, and MGIT was 71.0%, 74.2%, and 54.8%, respectively, with no significant differences among the three tests (*χ*^2^ = 3.000, *P* = 0.223). The sensitivity of MeltPro in BALF samples was similar to that in pulmonary puncture fluid samples (*χ*^2^ = 1.757, *P* = 0.185), and higher than that in sputum samples (*χ*^2^ = 17.050, *P* < 0.001).

**Fig. 3:**
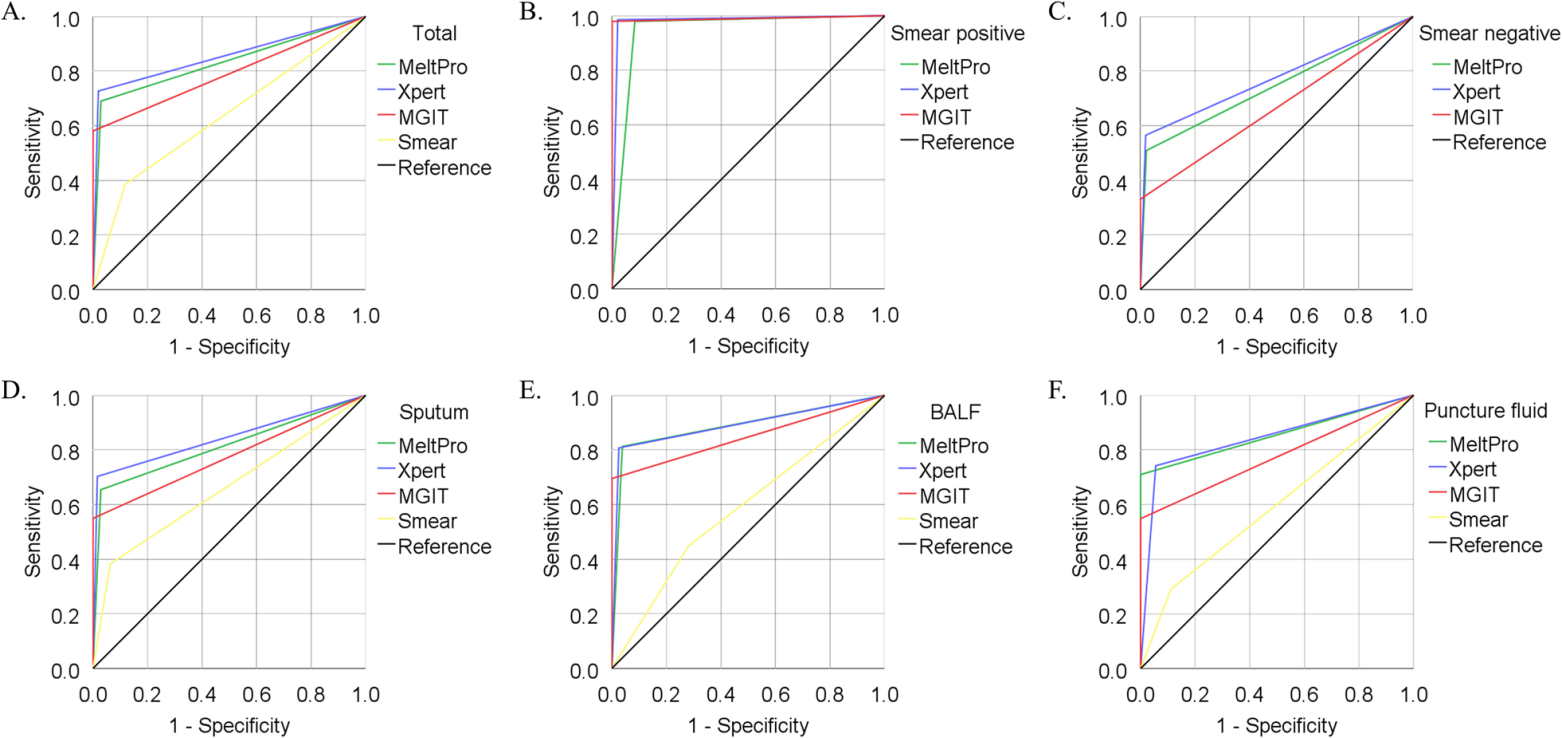
ROC curves representing the diagnostic performance of the tests for pulmonary tuberculosis diagnosis. Reference: The reference data includes cases finally diagnosed with pulmonary tuberculosis, encompassing bacteriologically confirmed, histopathologically confirmed, and clinically diagnosed cases. **A** Total cases (n = 1283); **B** Smear-positive cases (n = 385); **C** Smear-negative cases (n = 898); **D** Sputum samples (n = 969); **E** Bronchoalveolar lavage fluid (BALF) samples (n = 265); **F** Pulmonary puncture fluid samples (n = 49)

ROC curves of MeltPro, Xpert, MGIT, and smear for the diagnosis of PTB are shown in Fig. 3. The AUC of MeltPro, Xpert, MGIT, and smear in 1283 patients was 0.830, 0.854, 0.790, and 0.643, respectively. The AUC of MeltPro, Xpert, and MGIT in 898 smear-negative patients was 0.743, 0.771, and 0.660, respectively. In sputum or BALF samples, the AUC of MeltPro (0.813, 0.887) was lower than that of Xpert (0.844, 0.891). However, in puncture fluid samples, the AUC of MeltPro (0.855) was slightly higher than that of Xpert (0.843).

### Performance and ROC curves of MeltPro for detection of resistance to RIF, INH, EMB, STR and FQ

**Fig. 4:**
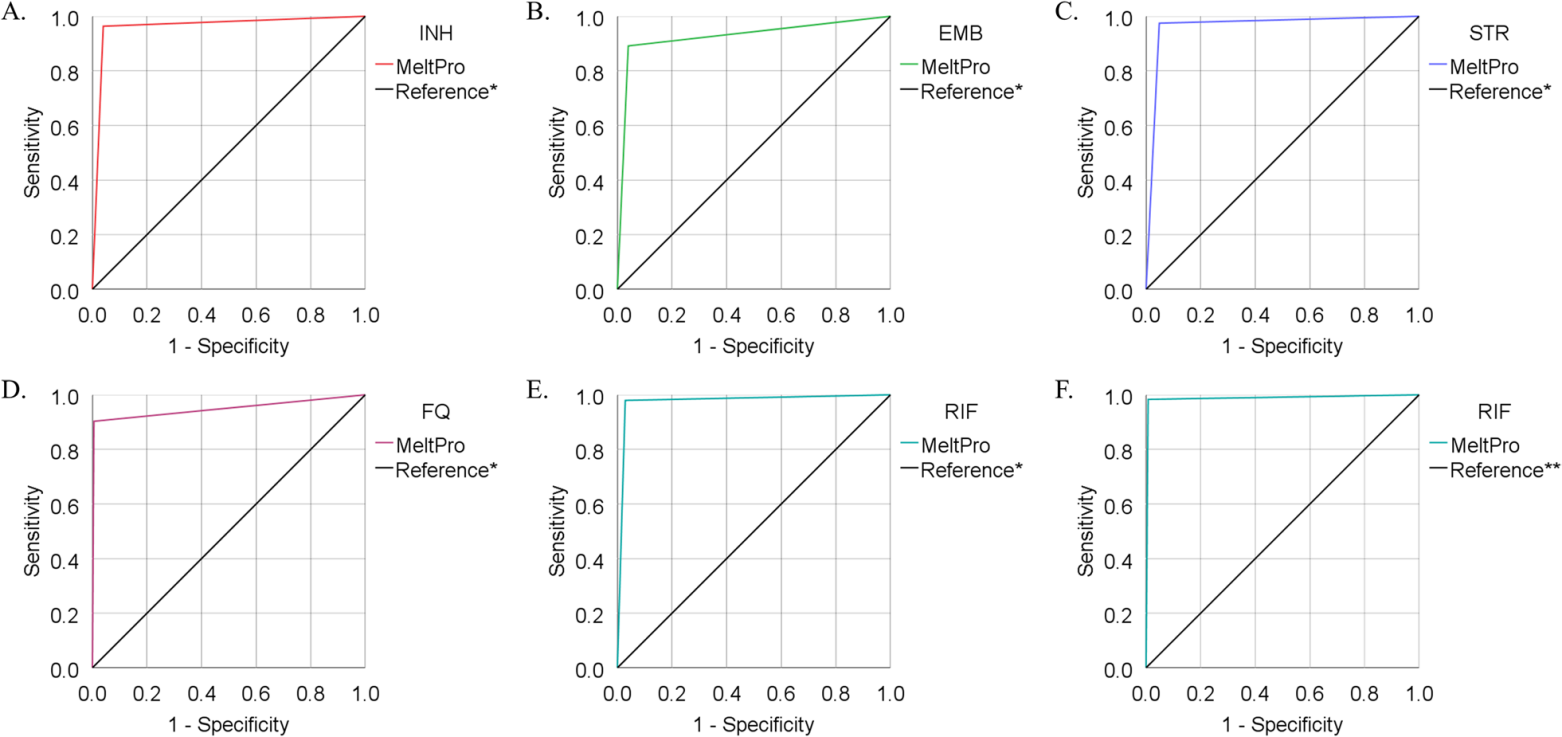
ROC curves displaying the diagnostic performance of MeltPro TB in detecting drug resistance. Reference*: Phenotypic drug-susceptibility testing results of MYCOTB. Reference**: Results of MYCOTB and/or Xpert MTB/RIF. **A** Resistance to isoniazid (INH) (n = 387); **B** Resistance to ethambutol (EMB) (n = 399); **C** Resistance to streptomycin (STR) (n = 391); **D** Resistance to fluoroquinolone (FQ) (n = 421); **E** Resistance to rifampicin (RIF) (n = 412); **F** Resistance to RIF (n = 517)

Based on the Xpert results, the sensitivity and specificity of MeltPro for detecting RIF resistance were 99.1% and 98.7%, respectively, with an AUC of 0.989 (95% CI: 0.978–1.000). Based on the MYCOTB results, the sensitivity and specificity of MeltPro for detecting RIF resistance were 98.0% and 97.1%, respectively, with an AUC of 0.975 (95% CI: 0.956–0.995). Based on the Xpert and/or MYCOTB results, the sensitivity and specificity of MeltPro for detecting RIF resistance were 98.9% and 99.0%, respectively, with an AUC of 0.988 (95% CI: 0.974–1.000). The results are listed in Table 3 and shown in Fig. 4.

**Table 3:** Diagnostic performance of MeltPro TB for detection of RIF resistance

Reference standard	Sensitivity % (95%CI; n/N)	Specificity % (95%CI; n/N)	PPV %	NPV %
MYCOTB	98.0 (92.1–99.7; 96/98)	97.1 (94.4–98.6; 305/314)	91.4	99.3
Xpert MTB/RIF	99.1 (94.6–99.9; 116/117)	98.7 (96.9–99.5; 385/390)	95.9	99.7
MYCOTB and/or Xpert MTB/RIF	98.3 (93.5–99.7; 118/120)	99.2 (97.6–99.8; 394/397)	97.5	99.5

*RIF* rifampicin, *PPV* positive predictive value, *NPV* negative predictive value

Based on the MYCOTB results, the sensitivity of MeltPro for detecting resistance to INH, EMB, STR, and FQ was 96.4%, 89.1%, 97.5%, and 90.3%, respectively. The specificity was 96.0%, 96.0%, 95.2%, and 99.4%, respectively. The AUC of MeltPro for detecting resistance to INH, EMB, STR, and FQ was 0.962, 0.926, 0.963, and 0.949, respectively. The results are listed in Table 4 and shown in Fig. 4.

**Table 4:** Diagnostic performance of MeltPro TB for detection of resistance to INH, EMB, STR, and FQ*

Drugs	Sensitivity % (95%CI; n/N)	Specificity % (95%CI; n/N)	PPV %	NPV %
INH	96.4 (90.4–98.8; 106/110)	96.0 (92.8–97.9; 266/277)	90.6	98.5
EMB	89.1 (75.6–95.9; 41/46)	96.0 (93.3–97.7; 339/353)	74.5	98.5
STR	97.5 (90.3–99.6; 77/79)	95.2 (92.0–97.2; 297/312)	83.7	99.3
FQ	90.3 (79.5–96.0; 56/62)	99.4 (97.8–99.9; 354/356)	96.6	98.3

*MYCOTB as reference standards; *INH* isoniazid, *EMB* ethambutol, *STR* streptomycin, *FQ* fluoroquinolone, *PPV* positive predictive value, *NPV* negative predictive value

## Discussion

Previous studies have evaluated MeltPro as a follow-on test for detecting drug resistance using cultured isolates (Hu et al. 2014), smear-positive specimens (Pang et al. 2016), and formalin-fixed, paraffin-embedded (FFPE) tissues from TB patients (Mu et al. 2021), and as an initial test for diagnosing spinal TB with pus specimens (Wang et al. 2018), and exhibited their reasonably diagnostic accuracy. In this study, we have evaluated the performance of MeltPro as an initial test for diagnosing PTB with drug-resistance detection. We used sputum, BALF, and pulmonary puncture fluid specimens from patients with presumptive PTB at two clinical centers. This is the first time such a retrospective and large sample analysis has been conducted.

Regarding the diagnosis of PTB, the overall sensitivity and specificity of MeltPro were 69.0% and 97.1%, respectively, while the overall sensitivity and specificity of Xpert MTB/RIF were 72.7% and 98.0% in the present study. There were no significant differences between the two tests. Xpert MTB/RIF is a WHO-approved molecular rapid diagnostic test and is recommended as an initial test for diagnosing TB and RIF resistance. A Cochrane Review on the diagnostic accuracy of Xpert MTB/RIF for PTB found a pooled sensitivity and specificity of 84.7% and 98.4%, respectively, using bacteriological culture as the reference standard (Zifodya et al. 2021). However, the sensitivity of Xpert MTB/RIF, based on a composite reference standard considering clinical and radiographic findings, was 72% in a previous study (Berhanu et al. 2018), which is similar to the sensitivities of MeltPro and Xpert in the current study, based on a reference standard of bacteriologically/histopathologically confirmed PTB and clinically diagnosed PTB.

In smear-positive patients, the sensitivity of MeltPro for diagnosing PTB was 97.9%, aligning closely with the WHO target product profile for this population (98%) (WHO 2021a). In smear-negative patients, both MeltPro and Xpert demonstrated similar sensitivities of 50.9% and 56.5%, respectively, in the current study. Previous research has shown that Xpert sensitivity in smear-negative, culture-positive participants ranges from 41 to 77% (Berhanu et al. 2018; Dorman et al. 2018; Mishra et al. 2020; Wang et al. 2019). The sensitivities of MeltPro for diagnosing PTB and smear-negative cases were observed to be suboptimal compared to Xpert in this study, implicating that MeltPro was incapable of identifying a greater number of true positive cases. The primary rationale behind this difference in detection capability could potentially be traced to the sample processing procedures. Notably, Xpert integrates DNA extraction, PCR amplification, and detection into a single self-enclosed test unit, and all steps are automated following sample loading (WHO 2021c). On the contrary, MeltPro involves a manual DNA extraction process preceding PCR and melting curve analysis, and this manual intervention may lead to incomplete DNA extraction and subsequent loss of DNA (Mu et al. 2021). Furthermore, the bacterial loads in smear negative patients are relatively low, thus true positive cases that cannot be identified mainly appear in these populations.

In this study, the sensitivity of MeltPro for diagnosing PTB in sputum, BALF, and pulmonary puncture fluid was 65.4%, 81.3%, and 71.0%, respectively; all of which were comparable to Xpert's performance. Prior studies have shown that both conventional detection methods and new molecular rapid diagnostic tests for PTB diagnosis in BALF specimens offer promising diagnostic potential compared to sputum specimens (Badr et al. 2022; Uddin et al. 2022). The current study's findings further support this notion, emphasizing the potential benefits of using bronchoscopy to collect BALF from patients with presumptive PTB for MeltPro testing to enhance diagnostic sensitivity. For patients with mass or nodular lesions, CT-guided percutaneous puncture of the lung was found to be a viable option for specimen collection.

Regarding the detection of RIF resistance, the sensitivity and specificity of MeltPro based on the MYCOTB results were 98.0% and 97.1%, respectively, in the current study. WHO recommends Xpert MTB/RIF as the initial test for detecting RIF resistance, and it has demonstrated high overall sensitivity (96%) and specificity (98%) when compared to phenotypic DST (WHO 2020). In the present study, based on the Xpert MTB/RIF and/or MYCOTB results, MeltPro's sensitivity and specificity increased to 98.3% and 99.2%, respectively. WHO also recommends moderate complexity automated NAATs as initial tests for detecting RIF resistance, showing overall pooled sensitivity and specificity of 96.7% and 98.9% (WHO 2021d), respectively. The data indicate that MeltPro performed with higher sensitivity as an initial test for detecting RIF resistance. The difference in phenotypic DST methods used may contribute to this variation, but the ability of MeltPro to detect heteroresistance is likely the major factor. A previous study demonstrated that a high melting curve assay can detect RIF resistance mutations down to a concentration of 5% mutant DNA (Van Rie et al. 2020), which is challenging for other tests to detect at such low levels of RIF heteroresistance.

In the present study, the sensitivity and specificity of MeltPro for detecting INH resistance were 96.4% and 96.0%, respectively, while the overall pooled sensitivity and specificity of moderate complexity automated NAATs were 86.4% and 99.2% (WHO 2021d), respectively. MeltPro's higher sensitivity may be attributed to its inclusion of the ahpC promoter region in addition to the inhA promoter region (17 to 8) and katG 315, which are the only two regions covered by moderate complexity automated NAATs. Previous studies have shown that mutations in the ahpC promoter account for 8.9–13% of total INH-resistant cases in China (Hu et al. 2014).

An important advantage of MeltPro is its ability to detect not only RIF and INH resistance but also resistance to FQ. According to the WHO's updated definition of pre-extensive drug-resistant tuberculosis (pre-XDR-TB) in 2021, it includes TB caused by M. tuberculosis strains that meet the criteria for multidrug-resistant or rifampicin-resistant tuberculosis (MDR/RR-TB) and are also resistant to any FQ (WHO 2020). A study on the prevalence of XDR-TB in a Chinese MDR-TB cohort after redefinition revealed that among a total of 425 MDR-TB isolates, 311 (73.2%) were FQ-resistant (Yao et al. 2021). These changes in definitions underscore the importance of detecting FQ resistance in high-burden countries like China. In this study, the sensitivity and specificity of MeltPro for detecting FQ resistance were 90.3% and 99.4%, respectively, while the overall pooled sensitivity and specificity of the Xpert MTB/XDR Assay (low complexity automated NAAT) were 93% and 98% (WHO 2021d), respectively, with MeltPro showing slightly lower sensitivity. It is worth noting that the low complexity automated NAAT is recommended by WHO as a follow-on test in specimens determined to be MTB-positive, but not as an initial test. In a study using sputum specimens from patients with smear-positive TB, the sensitivity and specificity of MeltPro for ofloxacin resistance against a reference standard based on phenotypic MGIT 960 DST were 83.3% and 98.1% (Pang et al. 2016), respectively. Several factors may account for the differences. Firstly, the frequencies of mutations conferring FQ resistance may vary between geographic regions, which could be the primary reason. Secondly, the relatively poor sensitivity of molecular methods in detecting heteroresistance compared to phenotypic DST methods (Torrea et al. 2019) might contribute to the variable performance of molecular methods in clinical samples, particularly in regions with high TB prevalence like China where FQ heteroresistance is relatively common (Hu et al. 2023). MeltPro also demonstrated high sensitivity (97.5%) and specificity (95.2%) for detecting STR resistance in this study. Hence, we suggest that MeltPro can serve as the initial test for detecting resistance to second-line injectable drugs in individuals with signs and symptoms of PTB.

It is worth noting that there could potentially exist other mechanisms contributing to drug resistance. Nevertheless, MeltPro possesses the capacity to exclusively identify resistance resulting from mutations encompassed within its assay. Furthermore, akin to other molecular tests, MeltPro focuses on the detection of nucleic acid sequences, rather than amino acid sequences. This approach means that even silent mutations, which do not lead to alterations in amino acids, might still identified as drug-resistant mutations.

In the current study, 12 MeltPro positive and 8 Xpert positive results were diagnosed as false positives based on the final diagnosis. Increased false positive results have been reported when using ultrasensitive molecular assays, such as Xpert, in individuals with a recent episode of tuberculosis (Huo et al. 2020). On the other hand, false positive results may arise in patients who have previously been treated with anti-TB drugs, given the fact that both Xpert and MeltPro cannot distinguish between alive and dead bacilli (Zainabadi et al. 2022).

Unlike Xpert, which utilizes a single self-enclosed real-time PCR test unit, MeltPro is compatible with nearly all mainstream real-time PCR machines. Consequently, MeltPro emerges as more convenient option compared to most other molecular tests, providing a shorter turnaround time for generating diagnostic results (David et al. 2023). Furthermore, the throughput of MeltPro exceeds that of Xpert in a single assay (96 vs 80), while its cost for detecting RIF and INH resistance is only half of Xpert's. The notable advantage of MeltPro lies in its capability to detect resistance to a broader range of anti-TB drugs, facilitating the timely initiation of an effective treatment regimen. This advantage position MeltPro as a viable alternative for the rapid simultaneous detection of MTB and drug resistance, particularly in resource-limited settings. However, the patient outcomes and cost-effectiveness associated with using MeltPro as an initial diagnostic test require further evaluation.

This study had several limitations. Firstly, although Xpert Ultra has a better TB detection capability (Chakravorty et al. 2017), we did not compare its diagnostic accuracy with MeltPro as Xpert Ultra has not yet been launched in the two centers of this study. Secondly, sequencing was not used to confirm the mutation types and to clarify discrepancies among MeltPro, Xpert, and MYCOTB. Thirdly, the fact that a proportion of patients diagnosed with PTB were culture-negative hindered further phenotypic DST analysis. Notably, this was a retrospective study conducted within the confines of two TB-specialized hospitals, which may inherently restrict the broader applicability of our results. Hence, a multicenter prospective study is deemed essential to affirm and validate the diagnostic performance of MeltPro.

## Conclusion

The MeltPro TB assay exhibited favorable performance as an initial diagnostic test for detecting MTB and drug resistance simultaneously in clinical samples from patients with presumptive PTB. Compared with Xpert, MeltPro showed a slightly lower sensitivity but similar specificity for the diagnosis of PTB. However, the advantage of MeltPro lies in its ability to simultaneously detect resistance to RIF, INH, FQ, STR, and EMB. The MeltPro TB assay could potentially be an effective alternative as an initial test for diagnosing PTB with drug-resistance detection in clinical practice. A multicenter prospective study is imperative to corroborate and substantiate the diagnostic performance of MeltPro.

## Data Availability

The datasets used and/or analyzed during the current study are available from the corresponding author on reasonable request.

## Abbreviations

AFB: Acid-fast bacilli
AUC: Area under the curve
BALF: Bronchoalveolar lavage fluid
DST: Drug-susceptibility testing
EMB: Ethambutol
FQ: Fluoroquinolone
INH: Isoniazid
MDR/RR-TB: Multidrug-resistant or rifampicin-resistant tuberculosis
MGIT: Mycobacteria growth indicator tube
MTB: *Mycobacterium tuberculosis*
MYCOTB: Broth microdilution plate method
NAAT: Nucleic acid amplification test
NPV: Negative predictive value
PPV: Positive predictive value
PTB: Pulmonary tuberculosis
RIF: Rifampicin
ROC: Receiver operating characteristic
STR: Streptomycin
TB: Tuberculosis
WHO: World Health Organization
XDR-TB: Extensive drug-resistant tuberculosis
